# Efficacy of acupuncture combined with oral Chinese medicine in the treatment of arrhythmia: A meta-analysis

**DOI:** 10.1097/MD.0000000000033174

**Published:** 2023-03-24

**Authors:** Sisi Ning, Lei Yan, Yan Li, Zhaoqiang Cui, Yun Wang, Jiawei Shi, Yuhong Zhao

**Affiliations:** a Department of Internal Medicine, Tianshan Hospital of Traditional Chinese Medicine in Changning District of Shanghai, Shanghai, China; b Department of Cardiology, Zhongshan Hospital, Fudan University, Shanghai, China; c Shanghai Institute of Cardiovascular Diseases, Shanghai, China.

**Keywords:** acupuncture combined with Oral Chinese Medicine, arrhythmias, meta-analysis, oral Chinese Medicine

## Abstract

At present, Western medicine treatment methods for arrhythmia emerge in an endless stream, but the accompanying side effects are also exposed, which brings pressure on medical resources and social economy. In recent years, the advantages of acupuncture combined with traditional Chinese medicine (TCM) in the control of arrhythmia have become increasingly prominent. Neiguan (PC6) is the collateral point in pericardium meridian; acupuncture at Neiguan can nourish the heart and calm the mind, and also plays an important role in treating arrhythmias. There is currently a lack of evidence-based medical evidence for the combination of acupuncture and TCM in the treatment of arrhythmia. This study aimed to investigate the effect of acupuncture combined with oral TCM in the treatment of arrhythmia.

**Methods:**

Randomized controlled trials published from the inception of databases to June 2022 were reviewed by searching the PubMed, Cochrane Library, Embase, CNKI, VIP, and WanFang databases. Review Manager 5.4.1 was used for the meta-analysis after the reviewers scanned the literature, extracted information, and identified the risk of bias.

**Results:**

Eleven randomized controlled trials with 804 patients were reviewed, including 402 and 402 patients in the treatment and control groups, respectively. The results of the meta-analysis showed a significant benefit of acupuncture plus oral TCM in terms of clinical effectiveness compared with oral TCM alone (n = 696; relative risk (RR), 1.22; 95% confidence interval (CI) 1.14 to 1.30; *P* < .00001) and in lowering the number of premature beats in 24 hours (n = 374; standard mean difference, −10,55; 95% confidence interval (95% CI) −14.61 to −6.49; *P* < .00001). Acupuncture plus oral TCM was also found to improve the conversion rate (n = 168; RR, 1.32; 95% CI, 1.14–1.52; *P* = .0002) and increase the left ventricular ejection fraction (n = 250; mean difference, 6.57; 95% CI, 4.11–9.04; *P* < .00001), but it had no significant increase in adverse events (n = 262; RR, 0.57; 95% CI 0.30–1.09; *P* = .09).

**Conclusion:**

Compared with oral TCM alone, acupuncture combined with oral TCM showed a clear benefit in treating arrhythmias and had no increase in adverse events.

## 1. Introduction

Cardiac arrhythmias mainly refer to irregularities in the frequency, rhythm, pacemaker, conduction velocity, or excitation sequence of the heart. Cardiac arrest is the most catastrophic emergency for those with arrhythmias, and approximately 3.7 million people die of sudden cardiac death each year.^[1]^ The current survival rate after a cardiac arrest is < 10%.^[2,3]^ Atrioventricular conduction abnormalities, atrial fibrillation, and ventricular tachyarrhythmias, defined as anatomical or functional inadequacies in the sinus node, are among the leading causes of death. Arrhythmia can be triggered by various clinical diseases including hypertension, diabetes, pulmonary illness, myocardial ischemia, myocardial infarction, and heart failure. Therefore, it is critical to identify the available therapeutic options for arrhythmias.

Traditional Chinese medicine (TCM) has been gradually embraced and encouraged by the worldwide community for decades, owing to its benefits of high safety and consistent efficacy in the treatment of numerous diseases.^[4,5]^ In addition, combining TCM with common Western medication can effectively minimize the side effects of Western medicine.^[6]^ Acupuncture, 1 of the most widely performed procedures in external interventions of TCM, has been established to be no less beneficial than antiarrhythmic drugs in the treatment of arrhythmias, such as atrial fibrillation, supraventricular tachycardia, and premature ventricular contractions.^[7,8]^ External acupuncture combined with oral administration of TCM has subsequently been shown in clinical trials to have a synergistic impact on clinical efficacy, which is superior to oral TCM alone.^[9,10]^ This study used a meta-analysis to assess the efficacy and safety of acupuncture combined with oral TCM for arrhythmia compared with oral TCM alone to provide evidence-based medical evidence.

## 2. Methods

This systematic review was conducted in accordance with the preferred reporting items for systematic reviews and meta-analyses guidelines. Ethical approval is not necessary for this review study.

### 2.1. Search strategy

The PubMed, Cochrane Library, Embase, CNKI, VIP, and WanFang databases were searched until June 2022. The queries utilized were determined by the specifics of each database, and the search strategy terms were as follows: CNKI was used as an example for the Chinese search strategy terms. (Subject: “ palpitation “ OR “ premature beat “ OR “ arrhythmia “ OR “ atrial fibrillation “ OR “ bradycardia “ OR “ tachycardia “) AND (Title/Keywords/Abstract”: acupuncture “ OR “ electropuncture “ OR “ warm acupuncture “) AND Title/Keywords/Abstract”: traditional Chinese medicine “ OR “ capsule “ OR “ granule “ OR “ prescription “）; PubMed is used as an example in a foreign language database. (“palpitation”[Title/Abstract] OR “premature beat”[Title/Abstract] OR “tachycardia”[Title/Abstract] OR “bradycardia”[Title/Abstract] OR “atrial fibrillation”[Title/Abstract] OR “arrhythmia”[Title/Abstract]) AND (“acupuncture”[Title/Abstract] OR “electropuncture”[Title/Abstract] OR (“warm”[All Fields] AND “acupuncture”[Title/Abstract]) AND “acupuncture”[Title/Abstract])) AND (“traditional Chinese medicine”[Title/Abstract] OR “prescription”[Title/Abstract] OR “decoction”[Title/Abstract] OR “granule” [Title/Abstract] OR “capsule” [Title/Abstract]).

### 2.2. Inclusion and exclusion criteria

The inclusion criteria were as follows: all patients were required to meet at least 1 of the current or previous arrhythmia diagnostic criteria, such as premature beats, atrial fibrillation, tachycardia, or bradycardia. In addition, the arrhythmia diagnosis criteria are included in the “Traditional Chinese Medicine Disease Diagnosis and Efficacy Criteria;” The control group received oral compound TCM with common antiarrhythmic drugs, and the study group was treated with acupuncture on the basis of the control group; All the studies should be randomized controlled trials (RCT), regardless of whether the allocation concealment and blind approach were adopted.

Studies were filtered out if they met 1 or more of the following exclusion criteria: case reports, systematic reviews, animal experiments, conference papers, etc; the control group received acupuncture or simple western medicine treatment; the data were published repeatedly or the data could not be extracted.

### 2.3. Outcome measures

Clinical efficacy of antiarrhythmia treatment is defined as either effective (including markedly effective and effective) or ineffective (including ineffective and aggravated), with markedly effective implying that clinical symptoms almost vanish, effective implying that clinical symptoms are significantly improved, and ineffective implying that symptoms do not improve or worsen. The clinical efficacy rate refers to the percentage of markedly effective and effective cases among the total number of cases. Primary outcome: Clinical efficacy rate; secondary outcomes: number of premature beats in 24 hours; conversion rate; left ventricular ejection fraction (LVEF); and adverse events.

### 2.4. Data extraction and assessment of quality

Two investigators individually extracted data based on the inclusion and exclusion criteria, and differences were resolved by discussion or consultation with a third party. For each qualified study, the following items were collected: basic information of included literature: author, publication year, sample size, and random sequence generation; study subjects with various cardiac arrhythmias; intervention measures: intervention methods and treatment courses in the study and control groups; and outcome measures.

The included studies were assessed using the Cochrane collaboration editor’s manual on the risk of bias assessment tool for RCTs. The evaluation items included random sequence generation, allocation concealment, blinding assessment, incomplete outcome data, selective reporting, and other bias. The results for each aspect were divided into 3 levels: low-risk, unclear, and high-risk. Two investigators independently assessed the quality of the study. If there was disagreement, a third examiner was asked to help reach a decision.

### 2.5. Statistical analysis

Statistical analysis was performed using the Review Manager 5.4.1 software (Cochrane handbook). The results were reported as the standard mean difference with a 95% confidence interval (95% CI) for continuous outcomes and relative risk (RR) with a 95% CI for dichotomous outcomes. Statistical heterogeneity among studies was analyzed using the *I*^2^ test and chi-square test. We used a fixed-effects model in the absence of heterogeneity (chi-squared test *P* value > .05, and the *I*^2^ test had a value < 50%). Otherwise, a random effects model was used. Subgroup and sensitivity analyses were performed to explore potential heterogeneities of the studies and to assess potentially confounding factors. Publication bias was detected using funnel plots.

## 3. Results

### 3.1. Literature search

**Figure 1.**
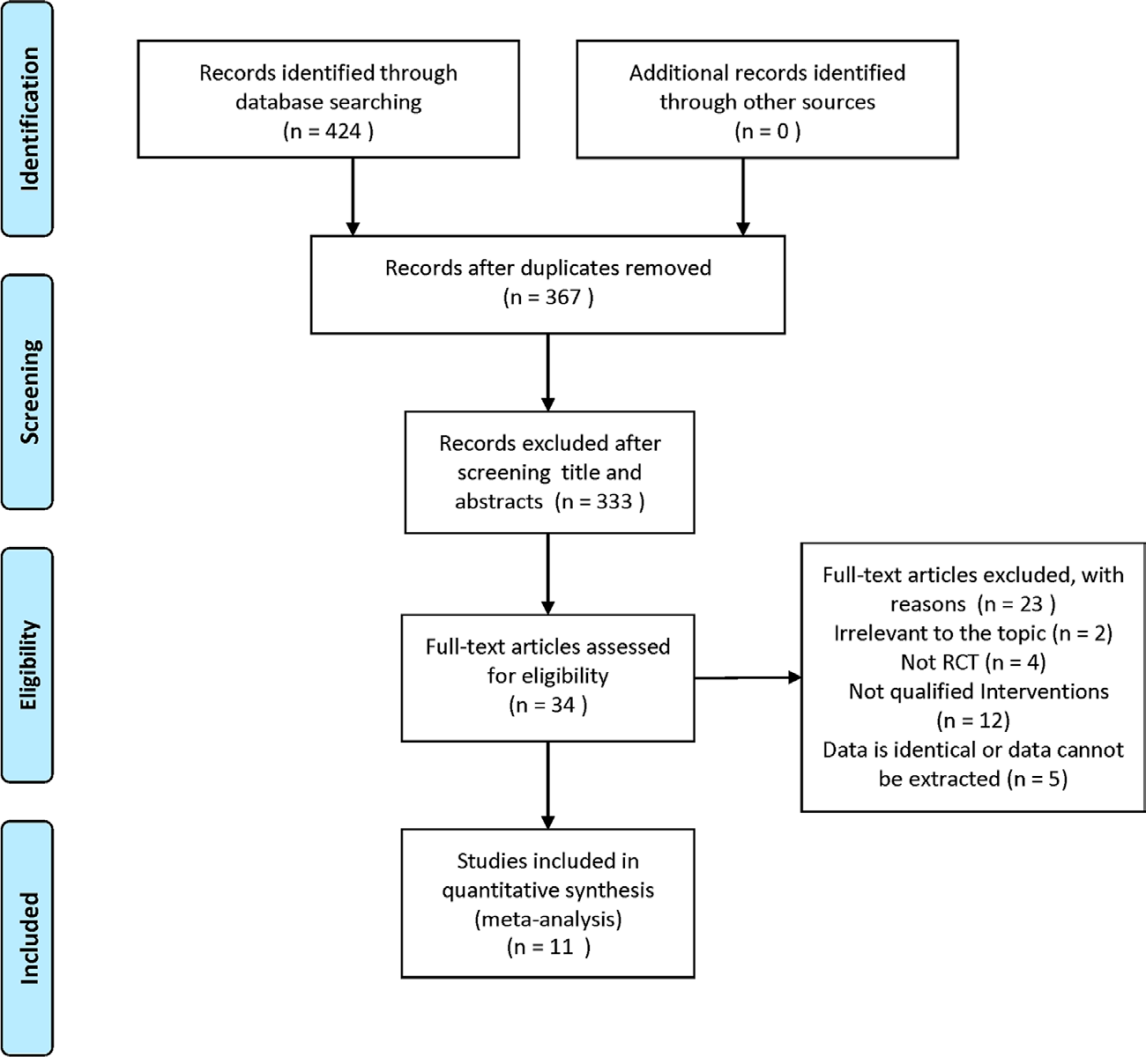
Flow Diagram.

In total, 424 articles were retrieved using the established search strategy. After removing 57 duplicates, 2 investigators reviewed 367 records for each title and abstract and a third investigator resolved any disagreements. Eleven RCTs with 804 patients^[9–19]^ were included in the meta-analysis. The flowchart in Figure 1 shows the complete selection and identification procedures.

### 3.2. Study characteristics

There were 11 RCTs^[9–19]^ in all, with 5 literature papers^[9–12,14]^ grouped by the random number table approach and the remaining 6 just mentioned randomization without clarifying the method. There was no mention of blinding or allocation concealment in any of the 11 studies. In addition to the TCM and acupuncture combined with TCM groups, there were other groups in 3 studies,^[10,14,19]^ but only the TCM and acupuncture combined with TCM groups were enrolled based on requirements. In 1 trial,^[14]^ patients were administered amiodarone, an antiarrhythmic medication, in addition to TCM or acupuncture plus TCM. Adverse events were reported only in 4 research.^[10,11,14,17]^ Adverse events included chest tightness, fatigue, pale complexion, and gastrointestinal reactions. All patients improved on their own or after symptomatic treatment and did not quit the trial. Table 1 summarizes the characteristics of the included studies.

**Table 1:** Summary of the included study characteristics.

			Number		Intervention			
References	Design	Randomization	Treatment group	Control group	Treatment group	Control group	Treatment course (w)	Outcomes
Yang 2017	RCT	Referring to a random number table	44	44	Qi-Invigorating and blood-activating Chinese herbs + acupuncture	Qi-Invigorating and blood-activating Chinese herbs	4	① ② ③
Ke 2016	RCT	Unclear	30	30	Yangxin decoction + acupuncture	Yangxin decoction	4	①⑤
Li 2020	RCT	Referring to a random number table	41	41	Gegen Guizhi Gancao decoction + amiodarone (0.2g qd) + acupuncture	Gegen Guizhi Gancao decoction + amiodarone (0.2g qd)	4	① ⑤
Ma 2018	RCT	Unclear	31	31	Pingxin Dingji decoction + acupuncture	Pingxin Dingji decoction	8	① ④
Li 2018	RCT	Unclear	30	30	Mahuang Fuzi Xixin decoction + acupuncture	Mahuang Fuzi Xixin decoction	2	① ⑤
Liu 2019	RCT	Unclear	50	50	Qi-Invigorating and blood-activating Chinese herbs + acupuncture	Qi-Invigorating and blood-activating Chinese herbs	4	① ② ④
Zhang 2013	RCT	Referring to a random number table	30	30	Wenxin Granules + acupuncture	Wenxin Granules	4	① ③⑤
Jin 2020	RCT	Referring to a random number table	60	60	Wenxin Granules + acupuncture	Wenxin Granules	8	① ②
Xu 2015	RCT	Referring to a random number table	54	54	Wenxin Granules + acupuncture	Wenxin Granules	2	③
Chen 2015	RCT	Unclear	33	33	Wenxin Granules + acupuncture	Wenxin Granules	4	① ②
Yuan 2012	RCT	Unclear	30	30	Wenxin Granules + acupuncture	Wenxin Granules	2	①

① Clinical efficacy rate; ② the number of premature beats in 24 hours; ③ Conversion rate; ④ left ventricular ejection fraction; ⑤ adverse events.

RCT = randomized controlled trials.

### 3.3. The risk of bias

**Figure 2.**
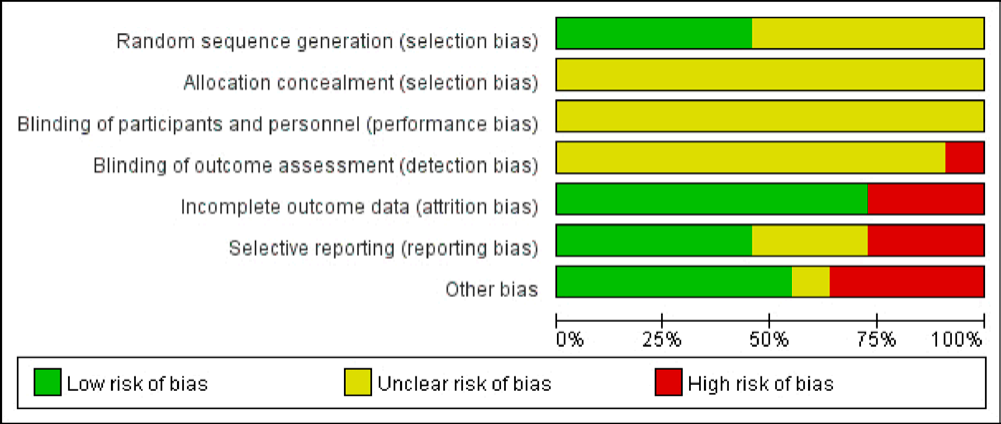
The risk of bias.

**Figure 3.**
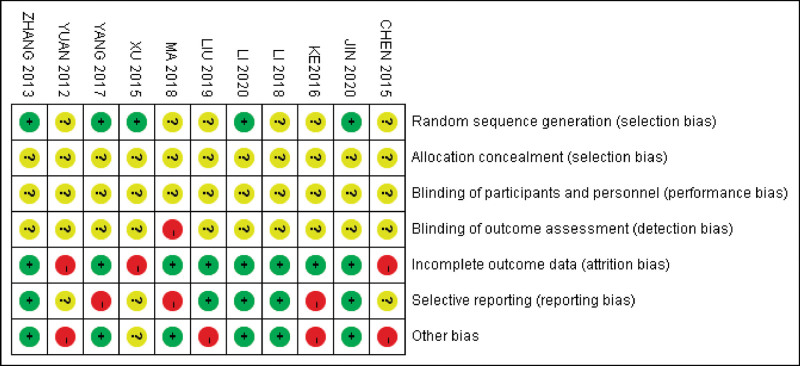
The risk of bias summary.

A review of the authors judgments about each risk of bias item is presented as a percentage across all included studies. The quality of the selected studies was assessed according to Cochrane criteria (Figures 2 and 3).

### 3.4. Primary outcome: Clinical efficacy rate

**Figure 4.**
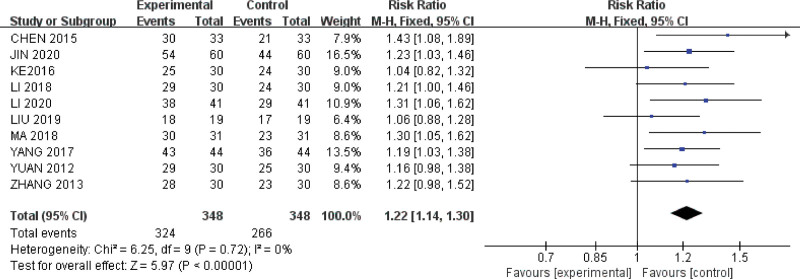
Meta-analysis of the clinical efficacy rate of acupuncture combined with TCM versus TCM alone for treating arrhythmia. TCM = traditional Chinese medicine.

Ten RCTs including 696 patients evaluated the clinical effectiveness of the intervention. No significant heterogeneity was observed among the studies (*I*^2^ = 0%, *P* = .72); therefore, the fixed-effects model was used for the meta-analysis. The pooled results showed that acupuncture combined with TCM was more effective than TCM alone in treating arrhythmia (RR, 1.22; 95% CI 1.14–1.30; *P* < .00001, Figure 4).

### 3.5. Secondary outcomes

#### 3.5.1. The number of premature beats in 24 hours.

**Figure 5.**
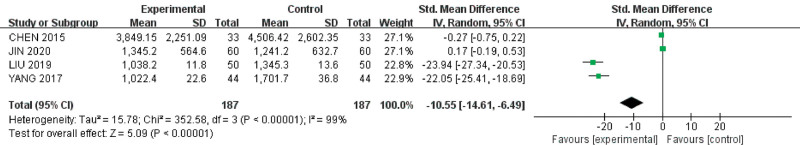
Meta-analysis of the number of premature beats in 24 hours of acupuncture combined with TCM versus TCM alone for treating arrhythmia. TCM = traditional Chinese medicine.

Compared to oral TCM, acupuncture coupled with TCM reduced the number of premature beats within 24 hours. (standard mean difference, −10,55; 95% CI −14.61 to −6.49; *P* < .00001, Fig. 5). We performed a sensitivity analysis by discarding 1 piece of research in each turn, and there was no change in the forest plot. The results were not reversed, implying that the results were reliable.

#### 3.5.2. Conversion rate.

**Figure 6.**
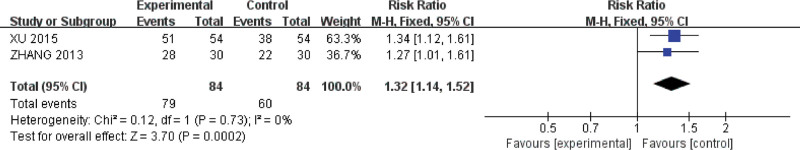
Meta-analysis of conversion rate of acupuncture combined with TCM versus TCM alone for treating arrhythmia. TCM = traditional Chinese medicine.

The result of the meta-analysis of these 2 RCTs revealed a statistically significant improvement of acupuncture plus oral TCM on the conversion rate for paroxysmal atrial fibrillation when compared to oral TCM alone. (RR, 1.32; 95% CI 1.14–1.52; *P* = .0002, Fig. 6). It shows that 2 RCTs can improve the conversion rate for paroxysmal atrial fibrillation.

#### 3.5.3. LVEF.

**Figure 7.**
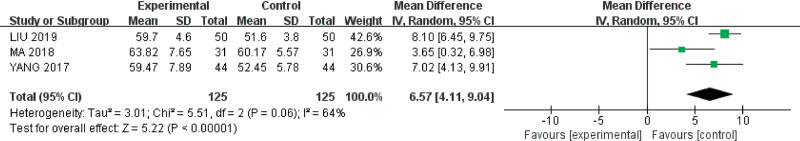
Meta-analysis of left ventricular ejection fraction of acupuncture combined with TCM versus TCM alone for treating arrhythmia. TCM = traditional Chinese medicine.

The results of the meta-analysis showed statistically significant differences in LVEF when treated by acupuncture plus oral TCM compared with oral TCM alone, while heterogeneity was shown in the included research (mean difference, 6.57; 95% CI 4.11–9.04; *P* < .00001, Fig. 7). According to the sensitivity analysis, Ma study was the cause of heterogeneity. It was included in the meta-analysis as it met the inclusion criteria, However, exclusion of this study did not have an effect on the pooled effect estimate or on statistical heterogeneity. Analysis above indicates that 3 RCTs can promote the LVEF in patients with arrhythmia.

#### 3.5.4. Adverse events.

**Figure 8.**
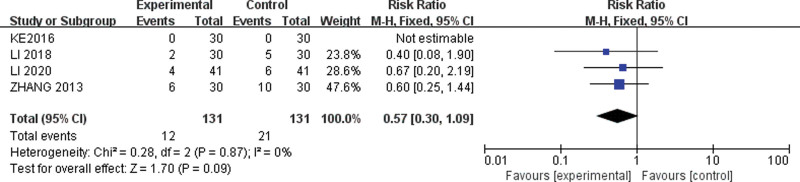
Meta-analysis of adverse events of acupuncture combined with TCM versus TCM alone for treating arrhythmia. TCM = traditional Chinese medicine.

The results of the meta-analysis showed no statistically significant difference in the adverse events of acupuncture plus oral TCM compared to oral TCM alone for arrhythmias (RR, 0.57; 95% CI 0.30–1.09; *P* = .09; Fig. 8). This also suggests that acupuncture combined with oral TCM had no significant increase in adverse events for treating patients with arrhythmia.

### 3.6. Publication bias

**Figure 9.**
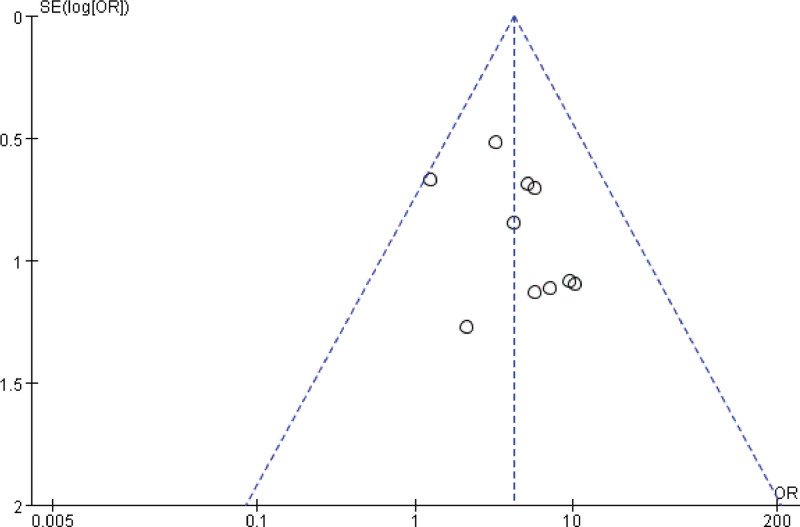
Funnel plot for publication.

The analysis of clinical effectiveness was done by Revman 5.4.1 to make a funnel plot. The scatter distribution is not completely symmetrical, as shown in Figure 9, indicating that there may be publication bias; however, as the number of included studies was small, the results might not be completely accurate.

## 4. Discussion

Our meta-analysis revealed that the clinical effectiveness of acupuncture combined with oral TCM was superior to that of oral TCM alone in patients with arrhythmia. In addition, internal and external therapies have more substantial effects in lowering the number of premature beats within 24 hours and raising cardiac ejection fraction when compared to traditional Chinese medicine’s simple oral administration. There were no significant differences in adverse events between the 2 treatments. According to previous research, acupuncture may improve the clinical efficacy of patients with arrhythmia without increasing the incidence of adverse events.

Both tachyarrhythmia and bradyarrhythmia were included in the meta-analysis. Under the compatibility theory of monarch, minister, assistant, and guide, various Chinese herbal medicines give full play to their efficacy and achieve the effect of treating both rapid and slow arrhythmia through multi-target, multi-path, multi-ion channel blocking, and nonion channel regulating mechanisms.^[20]^ The TCM prescriptions included in the study followed the treatment principles of TCM for palpitations, including nourishing qi and yin, promoting blood circulation and dredging collaterals, warming the kidney, and helping yang, reflecting the principle of treating the same disease with different methods in traditional Chinese medicine. Wenxin Granules, an antiarrhythmic Chinese patent medication, have been shown in a large-sample multi-center clinical study to have a clinical efficacy of more than 80% in the treatment of atrial premature beats and ventricular premature beats, with good safety during treatment.^[21]^ There are various ways to treat arrhythmia with TCM, and oral TCM has been widely used in the treatment of arrhythmia.

The treatment groups in 11 studies were administered conventional acupuncture therapy. It is not difficult to discover that PC6 Neiguan is the primary point for treating arrhythmia. The domestic physiology laboratory^[22]^ confirmed that the antiarrhythmic effect of acupuncture at the Neiguan acupoint decreases after the median nerve and its upper nerve roots are broken along the meridian where the Neiguan acupoint is located, proving that the antiarrhythmic action of the PC6 acupoint is related to the neuroendocrine system. Network correlation, while suggesting that the regulation of heart rate by acupuncture is predicated on a functional central nervous system.^[23]^ Sympathetic nerve and cardiovascular activities are coordinated by the cardiac sympathetic afferent reflex. Arrhythmia stimulation impulses reach 1 of their important structures, the nucleus of the solitary tract (NTS), which triggers neurons and promotes c-Fos cell expression. Animal experiments^[24]^ have shown that electro-acupuncture at the PC6 Neiguan acupoint can considerably reduce the number of c-Fos-positive cells. When the afferent information from acupuncture at the PC6 Neiguan acupoint, as well as the stimulation information from tachyarrhythmia or bradyarrhythmia, were transmitted into the NTS, they were integrated and reemitted to the heart, resulting in the recovery of cardiac rhythm. An animal experiment^[25]^ showed that point specific electro-acupuncture stimulation inhibits phenylbiguanide-induced vasodepression and bradycardia responses, PC6 Neiguan was 1 of the acupoints. They verified that this performance was achieved through a μ-opioid mechanism in NTS. A study of electro-acupuncture pretreatment at the PC6 Neiguan acupoint on myocardial ischemia reperfusion injury rats revealed that the arrhythmia score of rats pretreated with electro-acupuncture at the PC6 Neiguan acupoint was significantly decreased when compared with the rats that were not pretreated. In addition, electro-acupuncture pretreatment at the PC6 Neiguan acupoint could regulate oxidative stress and myocardial contraction-related signaling pathways in rats.^[26]^ This is also 1 of the reasons for the improvement in cardiac function in the acupuncture plus oral Chinese medicine group compared with the simple Chinese medicine group. The analysis of adverse events revealed that combining acupuncture with Chinese medicine did not increase the clinical risk of TCM treatment of arrhythmia, and that acupuncture and TCM therapies are simple and inexpensive, with a much lower incidence of adverse events than conventional antiarrhythmic drugs.

The limitations of this study include the following: the random sequence generation was primarily based on random number tables, and there was also unclear randomization in many studies. Blinding was not stated in any of the included trials; however, because acupuncture differs from conventional oral medicines, it is difficult to accomplish blinding in clinical applications. There is no standardization of the treatment course, acupoints, and needling manipulation for which acupuncture should be applied during research. There was a lack of long-term curative effect data in any of the included investigations, and the length of follow-up time and the rate of arrhythmia recurrence could not be overlooked when assessing the actual efficacy of acupuncture plus Chinese medicine. The findings of these clinical studies are still under discussion. The LVEF results showed high heterogeneity (I^2^ = 64%). According to the sensitivity analysis, Ma et al ‘s study was the cause of heterogeneity, which could be attributed to the following reasons. First, only 3 studies were included in this meta-analysis, which may have led to high heterogeneity. Second, the treatment course in Ma study differs from the other 2, as do the acupuncture endpoints. Third, the Chinese herbal formula received by the patients in Ma et al study was different from the other 2. Sensitivity analysis performed by removing the literature individually showed that there was no change in the forest plot. The results were not reversed, implying that the results were reliable. As a result, we saved and analyzed this study. This review, however, provides the existing level of evidence supporting acupuncture treatment for cardiac arrhythmias and offers a valuable understanding of acupuncture interventions for future clinical trials.

In summary, our meta-analysis demonstrated that combining acupuncture with oral Chinese medicine shows a clear benefit in treating arrhythmias and has no increased risk of adverse events. However, RCTs with large-sample sizes and rigorous designs should still be conducted, in addition to the adoption of long-term follow-up results, to help reduce bias.

## Author contributions

**Conceptualization:** Sisi Ning.

**Data curation:** Sisi Ning, Yun Wang, Jiawei Shi.

**Formal analysis:** Sisi Ning.

**Methodology:** Lei Yan, Yan Li, Zhaoqiang Cui.

**Software:** Zhaoqiang Cui, Yun Wang, Jiawei Shi.

**Supervision:** Yuhong Zhao.

**Writing – original draft:** Sisi Ning.
